# Synthetic spectral libraries for Raman model calibration

**DOI:** 10.1007/s00216-025-05985-y

**Published:** 2025-07-08

**Authors:** Louis V. Hellequin, Vicent J. Borràs, Patrick Romann, Nandita Vishwanathan, Jonathan Souquet, Thomas K. Villiger

**Affiliations:** 1 University of Applied Sciences Northwestern Switzerland FHNW, Muttenz, Switzerland; 2 RWTH Aachen University, Aachen, Germany; 3 Levitronix, Zurich, Switzerland; 4 Biotech Process Sciences, Merck KGaA, Corsier-sur-Vevey, Switzerland

**Keywords:** IR spectroscopy/Raman spectroscopy, Modeling, Synthetic data, Process analytical technology

## Abstract

Raman spectroscopy has become increasingly popular in the process analytical technology (PAT) landscape due to its versatility and predictive capability in bioprocesses. However, model building remains a time-consuming and cost-intensive task. Building upon a fast calibration workflow based on physical pure compounds spiking in water, this work explores the novel use of in silico spiking of pure spectral fingerprints of various analytes. Through data fusion, a synthetic spectral library (SSL) is created that combines base spectra information from mammalian cell culture runs with matrix variability, as well as pure component spectra in water, aiming to greatly reduce the cost and time required for efficient model building. The findings indicate that the in silico addition of pure compounds provides spectral information comparable to physically spiked measurements. Consequently, this approach allows for the generation of an extensive number of information-rich spectra, forming a robust foundation for various regression algorithms and enhancing Raman calibration of existing spectral databases.

**Graphical Abstract:**

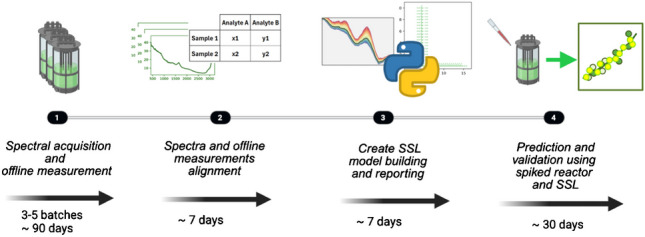

**Supplementary Information:**

The online version contains supplementary material available at 10.1007/s00216-025-05985-y.

## Introduction

Process analytical technology (PAT) has evolved into a cornerstone of the biopharmaceutical industry, enabling real-time monitoring, precise control, and enhanced process understanding in both batch and continuous manufacturing [1, 2]. Among the various analytical techniques available, vibrational spectroscopy such as Raman or infrared (IR) spectroscopy has gained significant attention due to its non-destructive nature, rapid analysis, and minimal sample preparation requirements [3]. With its low water interference, Raman spectroscopy provides critical insights into the molecular composition and dynamic changes occurring within bioprocesses, making it a versatile analytical tool for a wide array of applications, ranging from upstream fermentation [4] and downstream purification [5] to drug product processes [6]. Incorporating such PAT tools enables in situ measurements of several critical components in real-time, thereby enabling enhanced process understanding as well as real-time control, leading to consistent product quality and increased productivity [7]. However, the full potential of Raman and other spectroscopic techniques in industrial applications is often hindered by the challenges associated with their calibration.

Calibration of Raman spectroscopic models is a complex and delicate endeavor primarily due to the necessity of effective preprocessing [8], baseline correction [9], normalization [10, 11], precise wavelength selection, and smoothing prior to modeling of various regression algorithms such as partial least square (PLS), neural network, or other regression techniques [12–14]. These steps, although essential, do not address the crucial aspect of spectral acquisition and concomitant analysis of offline measurements. For example, various machine learning regression algorithms were evaluated to correlate up to 20 different offline measurements [15]. Although several non-traditional regression algorithms showed superior performance compared to traditional PLS regression, only the prediction of glucose and lactate seemed to result in satisfactory predictions [15]. Furthermore, data augmentation methods have been explored to artificially enhance the dataset’s size and diversity by creating synthetic data [16–18]. This trend is increasing due to machine learning (ML) models requiring larger datasets than simpler models as PLS regressions [18]. These methods, while useful, cannot substitute for real empirical data and, more importantly, they do not address the issues related to real-time data acquisition during process monitoring.

The concept of just-in-time (JIT) learning and calibration, which involves updating models in real-time, is an intriguing solution yet encounters several practical difficulties [19, 20]. Implementing JIT requires sophisticated instrumentation and computational resources capable of processing and updating calibration models on the fly. Additionally, the automated update and validation of these models present significant regulatory challenges, necessitating advanced algorithms that can adapt to changing process conditions without human intervention. While promising, these implementations are resource-intensive and may not be feasible for all biopharmaceutical manufacturers.

Robotic platforms offer another potential solution by enabling high-throughput and consistent data collection. These platforms can operate continuously and generate large volumes of data, which can then be used to develop and refine spectroscopic models [21]. However, the deployment of robotic platforms is often associated with significant capital and operational expenditure. Smaller differences in fluid dynamics, such as variations in flow rates, sedimentation, and other hydrodynamic phenomena, can also influence the Raman signal in the miniatured flow chambers and create challenges for consistent data acquisition and interpretation. These factors contribute to the complexity of deploying robotic platforms effectively for Raman spectroscopy calibration.

A simple yet effective strategy to gather information-rich spectra involves the use of pure compounds dissolved in water. As stated by Lee and colleagues [22], most explicit models assume that the Raman spectra of a mixture are the linear combination of the spectra of the pure components of the same mixture, and the analyte concentrations are typically estimated using classical least squares fitting. Potential non-linear effects resulting from component interactions are considered negligible in typical aqueous solutions where analyte concentrations are in the low single-digit mass percentage range or below. Although explicit models have been used to estimate different analyte of interests (AOIs) in *Escherichia coli* bioprocesses [22] and in CHO cell culture supernatants [23], this procedure is challenging in such complex solutions containing many different molecules, which limits its accuracy. For example, explicit models do not consider mixture constraints, and the estimated ratios do not always add up to 100%. To overcome this issue, Muteki et al*.* [24] implemented the iterative optimization technology (IOT) for both Raman and NIR spectra for simple model calibration, although these applications were limited to finalized pharmaceutical products which did not involve live organism cultures [24, 25].

In cases where non-linear effects become relevant, indirect hard modeling (IHM) offers another innovative strategy for Raman spectroscopy calibration [26, 27]. This modeling uses parametrized peaks (position, intensity, width, and shape) to describe the pure components of the mixture. Despite its successful application to the monitoring of glucose and ethanol during yeast fermentations, the quality of the predictions is highly dependent on the peak functions and degrees of freedom selected by the user [28, 29].

In a bioprocessing context, spiking pure compounds into the media [30–32], directly into the process [33], into the process streams [34] or incorporating them into a harvest library for perfusion processes [35] has been explored. These methods enhance the information content of the spectra and thus provide a more holistic dataset for model calibration and validation. By introducing known quantities of pure compounds, it is possible to break the correlation of different analytes of interest and create a series of reference spectra that can be utilized to enhance the calibration dataset. Given that experimental Raman signal acquisitions may be perturbed by fluorescence interference, Rayleigh scattering, sample-induced artifacts (self-absorption, thermal effects, photo-degradation), environmental optical interference (cosmic rays, ambient light fluctuations, laser instability), and instrumental anomalies (detector noise, spectrometer drift, stray light), spiking pure compounds inherently account for these effects.

Nonetheless, these methods involve manual labor for each new process, as well as AOI, and precise measurements, introducing potential for human error. Furthermore, the practical challenges associated with handling pure compounds and ensuring that they are accurately and consistently spiked into the process must be considered. Despite the abundance of high-quality spectral data of many bioprocesses, the advancements in preprocessing, machine learning algorithms, data augmentation, robotics and spiking algorithms, Raman calibration, and model validation remain a time-consuming and resource-intensive task.

The aim of this work is to present a novel methodology that integrates pure compound in water spiking with existing spectral datasets. Unlike traditional spiking approaches where compounds are introduced into the individual processes, our approach focuses on measuring the characteristic fingerprints of pure compounds at various concentrations in water. These fingerprints are then incorporated to generate a synthetic spectral library (SSL). This process facilitates the creation of an extensive library of in silico Raman spectra for any bioprocess, significantly enhancing the spectral information content.

## Material and methods

### Perfusion culture process, monitoring, and control

A CHO-K1 cell line from Merck (Merck Serono SA, Corsier-sur-Vevey, Switzerland) producing a bispecific antibody was put into cultivation for a period of 21 days, during which a specifically designed expansion medium was used (Merck Serono SA, Corsier-sur-Vevey, Switzerland). The seeding density of the perfusion bioreactors (Labfors 5 Cell, Infors HT, Bottmingen, Switzerland) was 0.6·10^6^ cells/mL, the culture conditions were set to 36.5 °C and a dissolved oxygen setpoint of 50% (VisiFerm DO Arc, Hamilton, Bonaduz, Switzerland). A pH of 7.07 ± 0.17 (EasyFerm Plus Arc, Hamilton, Bonaduz, Switzerland) was controlled through CO_2_ sparging and a 1.1 M Na_2_CO_3_ addition. The working volume of the bioreactor was 2 L from day 0 onwards, and the perfusion rate was set at 1.3 reactor volume a day. The harvest rate was gravimetrically controlled to maintain a constant bioreactor weight. Bioprocessing levitating pumps PuraLev® i30 SU (Levitronix, Zurich, Switzerland) were used for the perfusion process, combined with polyether sulfone hollow fibers with a 0.22-μm pore size (Repligen, Waltham, MA, USA). Once the steady-state viable cell concentration setpoint was reached, an online biocapacitance probe (Incyte Arc, Hamilton, Bonaduz, Switzerland) controlled the bleed to keep a viable cell volume of 12% throughout the rest of the run.

### Hardware setup and data acquisition

A Multispec Raman (tec5 AG, Steinbach, Germany) with 785-nm laser excitation wavelength and 500-mW power output, combined with the software MultiSpec Pro II (tec5 AG, Steinbach, Germany), was used for spectral acquisition. The Multispec Raman device was equipped with a charge-coupled device (CCD) sensor with a spectral range of 69.8–3226.9 cm^−1^ (1044 pixels) and 70.0–3225.0 (every 1 cm^−1^) after interpolation. However, the probe included a high-pass filter, which limited the effective working range to 378–3225 cm^−1^. For the presented Raman measurements, during which spectral acquisition times were 12 x 20s, either a Raman Immersible Probe MSR M571 (tec5 AG, Steinbach, Germany) or an InPhotonics Raman probe (InPhotonics, Norwood, MA) were used with a stainless steel 316 L flow-cell (in-house, FHNW workshop, Muttenz, Switzerland) with a chamber volume of 0.95mL and a PG 13.5 thread. More informations about the flow-cell can be found in previous publications. The offline workflow consisted in the pumping of light-protected and heated (37 °C) harvest samples through the flow cell at a rate of 2 L/day, giving a residence time of 40 s, equating the harvest rate of the bioreactor runs. Pure components were added to harvested cell culture supernatant and measured using the aforementioned acquisition parameters as previously described [35]. The spectra acquisition of water as well as pure components in water was conducted correspondingly. Spectral acquisition of pure compounds in water was conducted with concentrations of 0, 0.5, 1, 1.5, 2, 2.5, 3, 4, 5, 6, 7, 8, 9, and 10 g/L.

### Perfusion culture datasets

We considered two different datasets in this study. The first dataset contains 155 Raman spectra from five different runs, including manual spiking of glucose and raffinose with known concentrations of these compounds (Figs. 6 and 7). The second dataset contains 358 Raman spectra from three different runs with manual spiking of glucose and lactate, again with known concentrations (Fig. 8).

### Partial least square (PLS) regression

All data analysis was performed with Python 3.12. For the standard approach, we pre-processed the spectra with the first derivative and second-order polynomial Savitzky-Golay filter with 31 window points followed by a standard normal variate (SNV). After preprocessing, the 450–1500 cm^−1^ and 2750–2950 cm^−1^ ranges of the spectra, where most of the bands of the considered analytes appear, were used to calibrate PLS regression models. The number of components was fixed to 2 for all PLS regression models for the sake of comparability.

### Data analysis workflow of synthetic spectral libraries (SSL)

SSL are generated by artificially simulating spectra of analytes in the harvest at different concentrations by combining spectra of pure analytes in water with representative spectra of the harvest. It is assumed that the analyte and harvest mixture is ideal and non-linear effects caused by molecular interactions are negligible as typical concentrations in mammalian cell cultures rarely exceed 10 g/L representing less than 1% wt [22]. To combine these spectra, it is essential to ensure that they are normalized to the same reference to account for any experimental conditions that may alter the Raman intensity (e.g., laser intensity, refractive index). Therefore, the normalization was carried out systematically with the averaged intensity between 3205 and 3215 cm^−1^ (near the intense symmetric O–H stretching water peak). After normalization, the normalized fingerprint ${F}_{A, c}^{N}$ of each analyte *A* at a specific concentration *c* can be either calculated by subtracting the normalized water signal:

1 $${F}_{A, c}^{N} = {S}_{A,c}^{N}-{S}_{\text{water}}^{N}$$

where ${S}_{A,c}^{N}$ and ${S}_{\text{water}}^{N}$ refer to the normalized spectra of the analyte in water and the normalized spectra of water, respectively. The fingerprints can be also calculated by considering any another pair combination of pure compounds:

2 $${F}_{A,\;\pm (c1-c2)}^{N} = {\pm (S}_{A,c1}^{N}-{S}_{A,c2}^{N})$$

where ${F}_{A, \pm (c1-c2)}^{N}$ are the two normalized fingerprints (${F}_{A, +(c1-c2)}^{N}$ and ${F}_{A, -(c1-c2)}^{N}$) of the analyte *A* at *c*1-*c*2 and *c*2-*c*1 concentrations, whereas ${S}_{A,c1}^{N}$ and ${S}_{A,c2}^{N}$ refer to the normalized spectra of the analyte in water at concentration *c*1 and *c*2, respectively. All possible pair combinations are considered to generate the fingerprints. After normalization, a synthetically spiked spectrum ${S}_{SSL}^{N} \left(A,c\right)$ with a variety of additional concentrations *c* of analyte *A* can be calculated as follows:

3 $${S}_{\text{SSL}}^{N} \left(A,c\right)= {S}_{H}^{N} + {F}_{A, c}^{N}$$

where ${S}_{H}^{N}$ refers to the normalized spectra of the harvest (process sample). It is worth noting that the fingerprint contributions can either increase or decrease the initial concentration of the analyte in the harvest spectra. Several analytes of interest can also be combined by simply adding up all normalized fingerprint spectra from analyte ${A}_{i}$ at concentrations ${c}_{k}$:

4 $${S}_{\text{SSL}} = {S}_{H}^{N} +\sum_{i,k}{F}_{{A}_{i},{c}_{k}}^{N}$$

From the in silico generated spectral database, the second-order polynomial Savitzky-Golay filter with a window size of 31 is applied. After preprocessing, the 450–1500 cm^−1^ and 2750–2950 cm^−1^ ranges of the spectra, where most of the bands of the considered analytes appear, were used to calibrate PLS models.

The number of generated spectra depends on the number of analytes considered (${n}_{A}$), the number of concentrations per analyte in water (${n}_{c}^{{A}_{i}}$), and the number of base spectra (${n}_{B}$). If each analyte is added independently, the total number of generated spectra (${n}_{S1}$) is:

5 $${n}_{S1} ={n}_{B}\sum_{i}^{{n}_{A}}\frac{{n}_{c}^{{A}_{i}}!}{\left({n}_{c}^{{A}_{i}}-2\right)!}+{n}_{B}$$

If the different analytes are combined, the total number of spectra (${n}_{S2}$) is even greater:

6 $${n}_{S2} ={n}_{B}\prod_{i}^{{n}_{A}}\frac{{n}_{c}^{{A}_{i}}!}{\left({n}_{c}^{{A}_{i}}-2\right)!} + {n}_{S1}$$

Depending on the initial concentration of the analytes in the base spectra, some of the generated spectra may have negative concentrations which are non-physical, so the final number of generated spectra (${n}_{FS}$) used is between $\frac{{n}_{S1}+{n}_{B}}{2}$ and ${n}_{S1}$ ($\frac{{n}_{S1}+{n}_{B}}{2} \le { n}_{FS1} \le { n}_{S1}$) and between $\frac{{n}_{S2}-{n}_{S1}}{{2}^{{n}_{A}}}+\frac{{n}_{S1}+{n}_{B}}{2}$ and ${n}_{S2}$ ($\frac{{n}_{S2}-{n}_{S1}}{{2}^{{n}_{A}}}+\frac{{n}_{S1}+{n}_{B}}{2} \le { n}_{FS2 }\le {n}_{S2}$) respectively for each case. It is worth mentioning that the number of spectra could be increased even further if, instead of considering pair combinations (see Eq. 6), larger combinations are considered (e.g., ${F}_{A, c1-c2+c3-c4}^{N} = {S}_{A,c1}^{N}-{S}_{A,c2}^{N}+{S}_{A,c3}^{N}{-S}_{A,c4}^{N}$).

### Signal-to-noise ratio (SNR)

Thes signal-to-noise ratio (SNR) was computed as follows:

7 $$\text{SNR} = \frac{s-\overline{b}}{{b }_{\text{rms}}}$$

where $s$ is the peak signal (± 3 cm^−1^), $\overline{b}$ is the average background value (2450–2550 cm^−1^), and ${b}_{\text{rms}}$ refers to the root mean square value of the background relative to the mean background.

### Root mean square error prediction (RMSEP)

The root mean square error prediction (RMSEP) was computed as follows:

8 $$\text{RMSEP} =\sqrt{\sum_{i=1}^{n}\frac{{({\widehat{y}}_{i}-{y}_{i})}^{2}}{n}}$$

where ${\widehat{y}}_{i}$ and ${y}_{i}$ are the predicted and observed values, respectively, and $n$ is the number of samples.

## Results

### Comparison of different workflows

**Fig. 1:**
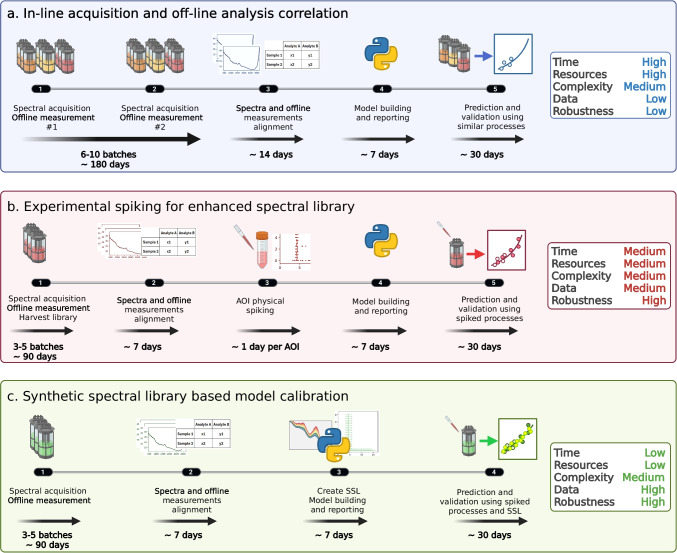
Schematic representation of different workflows. **a** The traditional approach where in-line spectral acquisition is timely aligned with the closest offline analysis requires data from several process runs for sufficient variability. **b** Experimental spiking can significantly reduce the amounts of experimental processes while concomitantly removing correlation of different analytes. **c** In silico spiking can generate similar information-rich spectra without any additional experimental effort

The two distinct Raman calibration methodologies employed in this manuscript together with the industry standard are illustrated in Fig. 1. The first approach, depicted in Fig. 1a and typically used in the industry, relies solely on spectral acquisition and offline analytics, necessitating multiple costly bioreactor runs to accumulate sufficient data for model development. This workflow can extend up to 6 months when accounting for the preparation of runs, sample allocation, and analysis along with signal processing. Furthermore, the data accrued for model building does not facilitate the decoupling of correlations between similarly varying analytes, resulting in suboptimal model performance. Testing and validation are often conducted with another batch that follows analogous overall trends. However, without physical spiking of the analyte of interest, model robustness may remain unachieved despite the apparent strong predictive power of the model, as the correlation between the process signal and the analyte signal has not been decoupled.

The second approach, conceptualized by Romann et al., capitalizes on the practical benefits of using frozen harvest library samples, avoiding the need for live runs to acquire qualitative data and enabling analyte spiking without disrupting an ongoing culture process, as shown in Fig. 1b [35]. Although this approach yields significant time savings and improved data quality, physical spiking must be performed individually for each process. It is noteworthy that the validation of chemometric models entails a spiking experiment at the conclusion, ensuring the model’s performance.

The third approach introduces a novel in silico spiking methodology termed SSL, which further extends time savings by measuring pure compounds in water at various concentrations (Fig. 1c). This method involves integrating these spectra with measurements from actual processes to impart the necessary matrix variation for optimal model performance.

Regardless of the chosen calibration approach, all regression models should be validated through physical spiking to ensure precise model calibration and reliability. In the following chapters, the results of the SSL approaches are presented, and ultimately, a comparative analysis of the three methodologies is conducted.

### Fingerprint of pure compounds in water

**Fig. 2:**
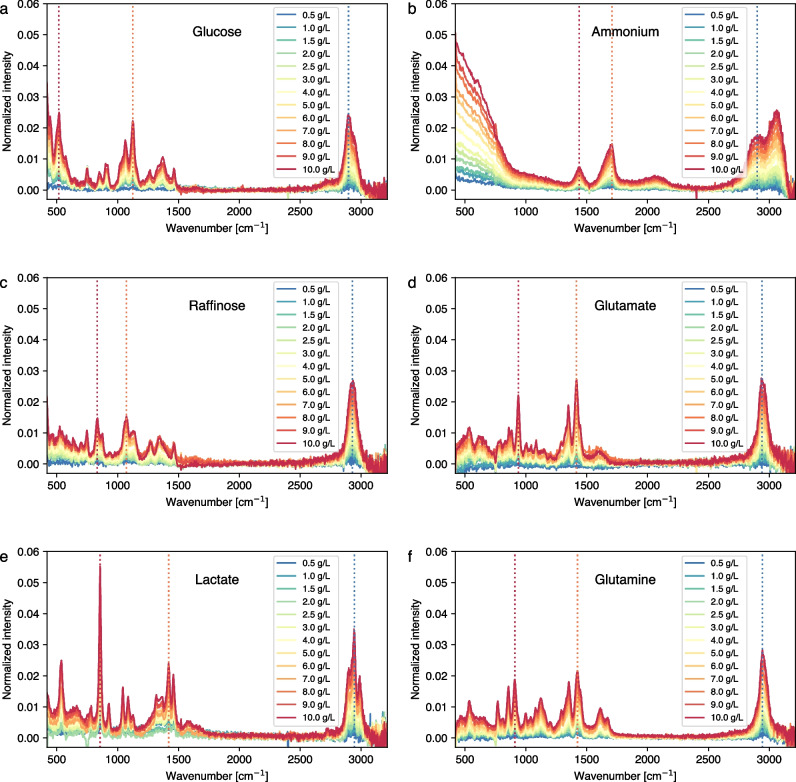
Normalized Raman spectra fingerprints of **a** glucose, **b** ammonium, **c** raffinose, **d** glutamate, **e** lactate, and **f** glutamine at 0.5, 1, 1.5, 2, 2.5, 3, 4, 5, 6, 7, 8, 9, and 10 g/L in water after subtraction of the Raman spectrum of pure water

The first step to generating the SSL is to measure the analytes in water at different concentrations. Figure 2 shows the Raman spectra of glucose, raffinose, lactate, ammonium, glutamate, and glutamine dissolved in water at different concentrations (0, 0.5, 1, 1.5, 2, 2.5, 3, 4, 5, 6, 7, 8, 9, 10 g/L). All spectra were normalized using the average signal between 3205 and 3215 cm^−1^ (near the intense symmetric O–H stretching mode of water). The water spectrum was subtracted to isolate the analytes’ contributions. In the spectra of the organic molecules (all but ammonium), two distinct regions can be observed: the lower part (up to ~ 1500 cm^−1^) where C–C and C-O modes appear, and the upper region (~ 3000 cm^−1^) where C-H and O–H modes are present.

Raffinose is a trisaccharide composed of glucose, galactose, and fructose. Although it is not normally used in cell cultures, it was selected as a worst-case scenario due to the significant overlap with glucose Raman spectra, as can be observed by comparing Fig. 2a and c. As demonstrated by Romann et al. [35], PLS models must be calibrated with spectra containing sufficient concentration variability of both analytes with overlaps. In addition, physical spiking during the test run is recommended to demonstrate that the models can properly distinguish both analytes. The measured fingerprints for the present study are in agreement with other previously published works [30, 33, 34].

**Fig. 3:**
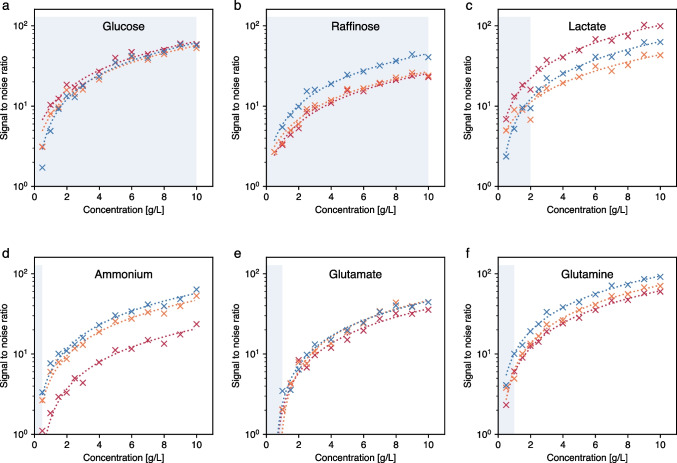
Signal-to-noise ratio (SNR) of **a** glucose, **b** raffinose, **c** lactate, **d** ammonium, **e** glutamate and **f** glutamine for each of the three peaks marked with vertical dashed lines in Fig. 2. The colors (blue, red, and orange) correspond to the vertical lines in Fig. 2. A regression line is added for each peak and analyte as a guide. The shaded area indicates the typical concentration range in mammalian bioprocesses

To compare the feasibility of detecting these analytes using the Raman measurement scheme described in the “Material and methods” section, Fig. 3 shows the SNR for three bands of each analyte (dashed lines in Fig. 2). The linear representation of the SNR as a function of analyte concentration is provided in the supplementary information (Figure SI 1). The SNR in Raman measurements is predominantly influenced by factors such as laser power and wavelength, integration time, sample properties, detector sensitivity, and optical configuration. Optimizing these parameters enhances the Raman signal intensity and reduces background noise, culminating in improved accuracy and reliability of the measurements. Although all analytes show similar trends in SNR, the shaded regions in Fig. 2 reveal that the typical concentrations of certain analytes (ammonium, glutamate, and glutamine) in mammalian bioprocesses fall in the lower range, where SNR is below 10. In such cases, the Raman model calibration and prediction is challenging, indicated by several publications [35, 36]. Since the optimization of SNR is beyond the scope of this manuscript, we will focus on the more detectable analytes (glucose, raffinose, and lactate) hereafter.

### Synthetic spectral libraries (SSL)

**Fig. 4:**
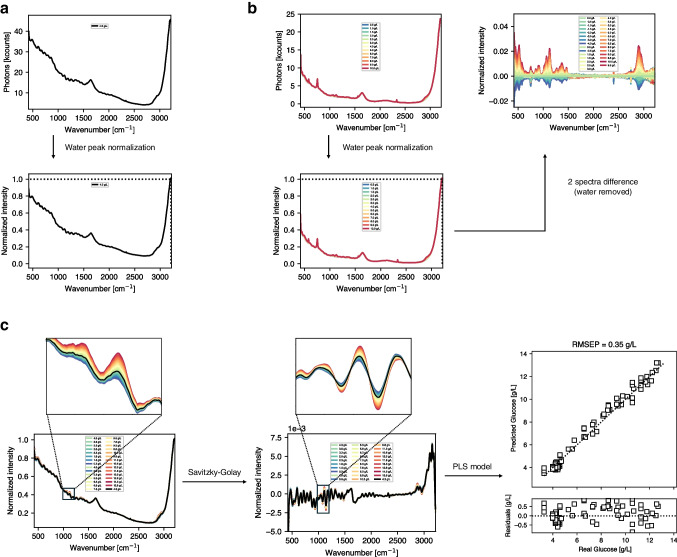
Overview of generation of synthetic spiked spectral libraries: **a** all raw Raman spectra are transformed into normalized Raman spectra. **b** Similarly, raw Raman spectral data from pure compounds in water were also normalized. After subtracting the signal of pure water, the normalized fingerprints of pure compounds are obtained, illustratively shown for glucose in water at 0.5, 1, 1.5, 2, 2.5, 3, 4, 5, 6, 7, 8, 9, and 10 g/L. **c** A single normalized Raman spectrum was synthetically augmented by adding to the normalized harvest spectrum for illustrative purposes. Then the first derivative and second-order polynomial Savitzky-Golay filter with a window of 31 points was applied. After performing the same procedure with 15 different base spectra, the synthetic spectral library (SSL) is generated that builds the basis for PLS regression

The Raman measurements of the different analytes in water can now be combined with representative bioprocess harvest spectra to generate SSL for modeling. Figure 4 illustrates this methodology by combining glucose measurements in water with a single harvest spectrum to simulate different glucose concentrations in the harvest. First, all spectra are normalized to the averaged intensity between 3205 and 3215 cm^−1^, around the O–H stretching mode of water (Fig. 4a, b). The differences between glucose concentrations are then used to determine the contribution of glucose at various concentrations (e.g., the contribution of 2 g/L glucose can be calculated as 2 g/L glucose – water, 4 g/L – 2 g/L) (Fig. 4b). Note that these contributions can lead to negative normalized intensities, as all possible combinations are performed, as stated in Eq. 2. These contributions are then added to the harvest spectrum to generate the synthetically spiked library (Fig. 4c). Finally, the spectra are processed with a first derivative Savitzky-Golay filter with a window size of 31 and a second-order polynomial before PLS regression.

### Comparison of physically and synthetically spiked Raman spectra

**Fig. 5:**
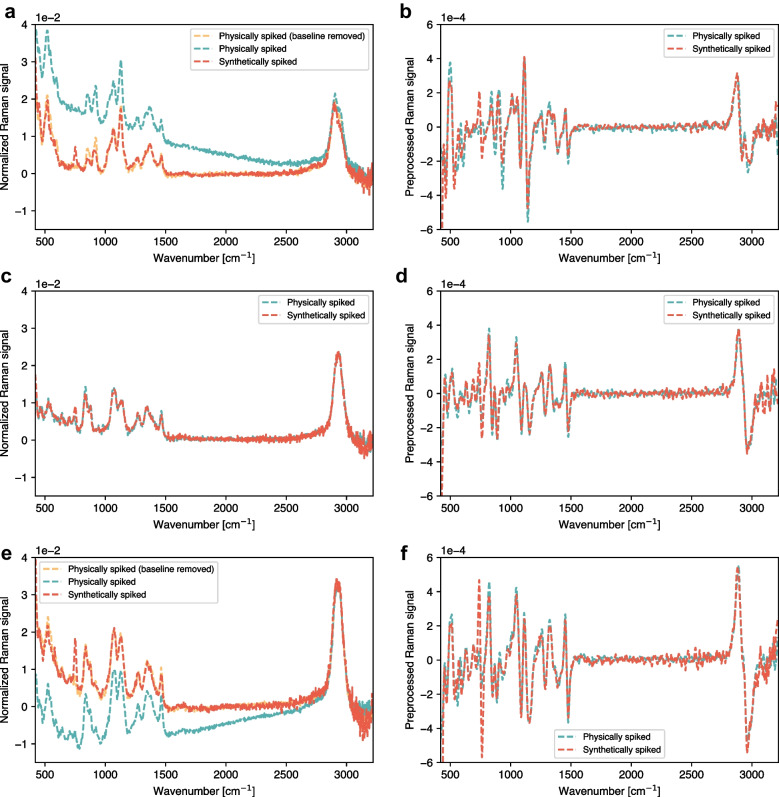
Comparison of physically and synthetically spiked Raman spectra. **a** Physically and synthetically spiked spectra referenced to the base spectrum with 8 g/L of glucose. The baseline was fitted with a third-order polynomial and removed for the physically spiked spectra. **b** The preprocessed Raman signal with the first derivative and second-order polynomial Savitzky-Golay filter with 31-point windows of the same spectra. **c** The comparison of the physically and synthetically spiked Raman spectra for 9 g/L of raffinose and **d** the corresponding signal after preprocessing. **e** The comparison of the physically and synthetically spiked Raman spectra for 4 g/L glucose and 10 g/L of raffinose and **f** the corresponding processed signal

Before evaluating the performance of the SSL library in training a prediction model, we first compared the spectra generated in silico and the spectra from physically spiked samples. Figure 5a, c, and e show a comparison of the in silico generated spectra and the experimental spectra, whereas Fig. 5b, d, and f illustrate these spectra after preprocessing. The spectra are referenced to the base spectrum for better visualization. In Fig. 5a, 8 g/L of glucose was synthetically spiked via SSL (red dotted lines) or physically spiked (green dotted lines), respectively. These baselines are not always identical and are most likely due to experimental variations between the base and spiked measurements such as small laser variations and different temperatures during measurements. No baselines are observed in the in silico spectra as the Raman measurements in water were carefully performed to obtain consistent spectra. To better compare the spectra with different baselines, we fitted them with a third-order polynomial and subtracted it. The agreement between the spectra is quite remarkable, with only small discrepancies observed around 750 cm^−1^ (Fig. 5b), demonstrating that the changes in the Raman spectra due to the addition of glucose and raffinose are consistent in both water and the harvest sample. This suggests that the Raman signal response to the addition of these analytes is mainly independent of the matrix (i.e., the harvest solution), as we assume for the generation of the SSL.

Ideally, the physically and synthetically spiked Raman spectra align even before processing as illustrated in Fig. 5b for 9 g/L of raffinose, resulting in comparable corresponding signal after preprocessing (Fig. 5d). However, it seems that the observed baseline shift does not significantly impact the comparison of the physically and synthetically spiked Raman spectra, demonstrated with another baseline shift in Fig. 5c and f for the case of two spiked analytes (4 g/L glucose and 10 g/L of raffinose) and the corresponding processed signal. Therefore, the conventional preprocessing algorithms appear to be effective for baseline correction.

**Fig. 6:**
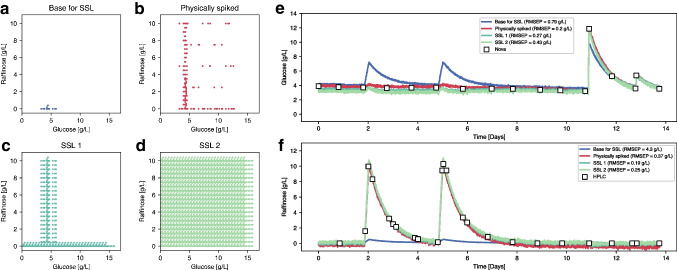
**a** Raffinose and glucose concentrations of 15 sample points from 5 different runs used as a base for creating the SSL. **b** Concentrations of the 155 spectra from physically spiked harvest samples. **c** Concentrations of the 3585 SSL1 spectra generated by in silico spiking of glucose and raffinose independently. **d** Concentrations of the 204,240 SSL2 spectra generated by in silico spiking of glucose and raffinose simultaneously. **e** Glucose concentration predictions of the verification run using PLS models calibrated with base (blue), physically (red), and synthetically (light and dark green) spiked spectra. **f** Raffinose concentration predictions of the verification run using PLS models calibrated with base (blue), physically (red), and synthetically (light and dark green) spiked spectra

Apart from the visual comparison presented in Fig. 5, synthetically and physically spiked spectra can be compared in terms of their correlation structure. To ensure that PLS models calibrated with synthetic spectra preserve the correlation structure of physically spiked spectra, we computed the DModX values (see Table SI 1) for the spectra shown in Fig. 5 using two PLS models calibrated with an SSL to predict glucose and raffinose respectively (see the “Raman model comparison between physical and synthetically spiked spectra” section and Fig. 6). The results obtained are below the maximum thresholds (99th percentile of the DModX values obtained for the calibration data), indicating that the physically spiked spectra lie within the correlation structure of the PLS models calibrated with synthetic spectra. In addition, Figure SI 2 shows the same spectra projected onto the latent space of the PLS models, revealing that the synthetically and physically spiked spectra appear close together in all cases. Thus, it can be assumed that the SSL spectra should be as effective as real spectra for model calibration.

### Raman model comparison between physical and synthetically spiked spectra

To ensure that the SSL data could be used to calibrate concentration prediction models and give results similar to those obtained with physical spiking, we tested it using the two different sets of measurements and analytes described in material and methods.

From the first dataset, 15 spectra of samples containing less than 6 g/L of glucose and less than 0.5 g/L of raffinose, as depicted in Fig. 6a, were used as the base for the SSL. Figure 6b shows the design of the experiment (DOE) matrix used during the physical spiking experiment. Figure 6 c and d indicate the concentration ranges reached by SSL1 and SSL2 generation, respectively, which differ by the addition of glucose and raffinose individually or with all different combinations possible (see Table 1 for more details). In Fig. 6e and f, the PLS models’ predictions are shown for glucose and raffinose concentrations, respectively, alongside offline measurements. To better observe the accuracy of the models, Figure SI 3 also presents the predicted versus real concentrations and residuals across the entire concentration range. The model generated from the base spectra represents the first modeling approach from Fig. 1a and failed to predict both analytes, especially raffinose. This failure can be explained by examining the concentration ranges of the calibration samples, which were nearly zero for raffinose and quite small for glucose (< 2 g/L). Additionally, since the model had not been trained on significant changes in raffinose concentration, it cannot properly distinguish between glucose and raffinose, increasing the glucose prediction when raffinose is added [35]. On the other hand, the other models produced similar results, with all being able to distinguish between glucose and raffinose and provide quantitatively accurate predictions (RMSEP < 0.4 g/L in all cases). For glucose, the model with physically spiked analytes produced slightly better results (RMSEP = 0.20 g/L) than SSL1 (RMSEP = 0.27 g/L). In contrast, for raffinose, SSL1 (RMSEP = 0.19 g/L) outperformed the physically spiked model (RMSEP = 0.37 g/L). In both cases, SSL2 produced slightly worse results than SSL1. Although the differences are likely not statistically significant, these results suggest that there is no significant improvement when generating spectra that combine the addition of both analytes.

**Table 1:** Number of base spectra (${n}_{B}$) and analytes (${n}_{A}$) used to generate the SSL. Maximum number of spectra that can be generated (physical and non-physical) when the fingerprints of the different analytes are added individually (${n}_{S1}$) or combined (${n}_{S2}$). The final number of spectra ${n}_{SSL1}$ and ${n}_{SSL2}$ are determined after removing the non-physical ones (negative concentrations)

	Number of base spectra	Number of analytes	Theoretical maximum number of individual spectra	Theoretical maximum number of spectra	Number of generated spectra for SSL1	Number of generated spectra for SSL2
	$n_B$	$n_A$	$n_{S1}$	$n_{S2}$	$n_{\mathbf S\mathbf S\mathbf L1}$	$n_{\mathbf S\mathbf S\mathbf L2}$
Gluc-Raf	15	2	5475	502,335	3585	204,240
Gluc-Raf 2	5	2	1825	167,445	1815	165,615
Gluc-Lac	15	2	5475	502,335	3645	211,500

**Fig. 7:**
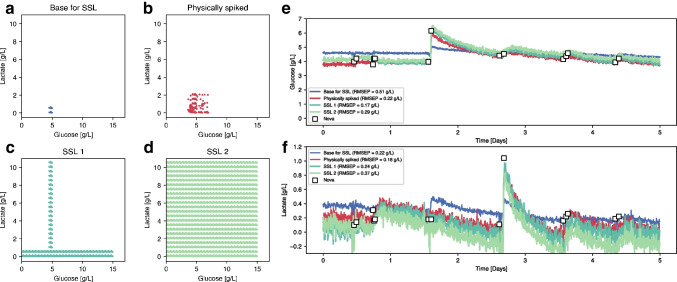
**a** Raffinose and glucose concentrations of 5 sample points from 3 different runs used as a base for creating the SSL. **b** Concentrations of the 155 spectra from physically spiked harvest samples. **c** Concentrations of the 1815 SSL1 spectra generated by in silico spiking of glucose and raffinose independently. **d** Concentration of the 165,615 SSL2 spectra generated by in silico spiking of glucose and raffinose simultaneously. **e** Glucose concentration predictions of the verification run using PLS models calibrated with base (blue), physically (red), and synthetically (light and dark green) spiked spectra. **f** Raffinose concentration predictions of the verification run using PLS models calibrated with base (blue), physically (red), and synthetically (light and dark green) spiked spectra

To confirm that the SSL works properly under conditions where the available spectra are fewer and less representative of the process to model but still produces similar results, a new set of models was generated for the first dataset using a completely different base set. This time, the base set contains only 5 samples from 3 different runs with > 8 g/L of glucose and 10 g/L of raffinose (see Fig. 7a), so there are three times fewer samples, less variability (3 runs instead of 5), and negative spiking becomes more relevant as starting concentrations are greater. Starting from this base set, two sets of samples were synthetically generated, expanding the concentration range to both lower and higher values (see Fig. 7c and d). In Fig. 7e, f and Figure SI 4, the predictions show similar results for the SSL1, SSL2, an physically spiked models (all RMSEP < 0.5 g/L). In contrast, the base predictions are significantly worse, especially for raffinose. This can also be explained by the fact that the concentration ranges are very small for glucose and 0 for raffinose (all base samples contain 10 g/L of raffinose).

**Fig. 8:**
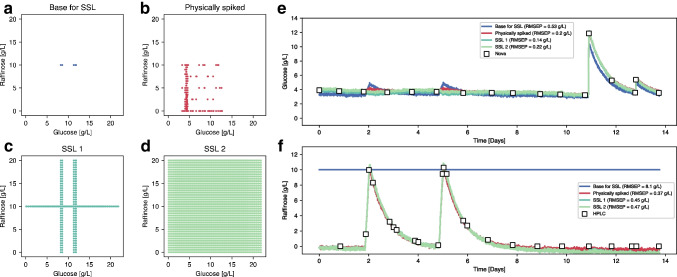
**a** Lactate and glucose concentrations of the 15 Raman spectra used as a base for creating the two SSL. **b** Concentrations of the 358 physically spiked harvest samples. **c** Concentrations of the 3645 SSL1 spectra generated by in silico spiking of glucose and raffinose independently. **d** Concentrations of the 211,500 SSL2 spectra generated by in silico spiking of glucose and raffinose simultaneously. **e** Glucose concentration predictions of the verification run using PLS models calibrated with base (blue), physically (red), and synthetically (light and dark green) spiked spectra. **f** Raffinose concentration predictions of the verification run using PLS models calibrated with base (blue), physically (red), and synthetically (light and dark green) spiked spectra

In the same fashion, the second dataset is composed of 15 spectra of samples containing between 4.5 and 5 g/L of glucose and less than 0.7 g/L of lactate (Fig. 8a). The DOE matrix for the physical spiking of glucose and lactate is presented in Fig. 8b. Similarly to the previous figure, Fig. 8 c and d illustrate the concentrations covered by SSL1 and SSL2 respectively, with individual or combined analytes. Predicted concentrations of glucose and lactate from the PLS models calibrated with each dataset are shown in Fig. 8e, f and Figure SI 5. Similar trends were observed across all models. The concentration ranges are too small in the base spectra to accurately predict and discriminate glucose and lactate. For glucose, this time, the SSL1 prediction (RMSEP = 0.17 g/L) outperformed the physically spiked one (RMSEP = 0.22 g/L). In contrast, for lactate, the physically spiked model gives a slightly better prediction (RMSEP = 0.18 g/L) than SSL1 (RMSEP = 0.24 g/L) and SSL2 (RMSEP = 0.34 g/L). In addition to the obvious fact that models need to be calibrated with samples containing a sufficient concentration range to produce accurate results, these findings also demonstrate that the SSL models perform similarly to the physically spiked models in two different runs and for three different analytes (glucose, raffinose, and lactate) irrespective of the initial concentrations of the base samples.

## Discussion

The standard workflow for preparing Raman calibration models for concentration prediction is time consuming and expensive as many different batches and offline samples need to be measured and aligned. Only minor discrepancies between physical and in silico generated spectra were found and models performed very comparably, whether physically spiked or in silico spiked spectra were used. Therefore, this novel approach has several key advantages.

First, during the development of the calibration models, the SNR of the analyte in water represents a best-case scenario indicating the feasibility of having a robust model calibration. In our setup, ammonium, glutamine, and glutamate have shown low SNR in the concentration ranges and thus calibration models based on the physical fingerprints are difficult to establish. In contrast, robust and accurate models can be established when molecules are detected in the area where SNR is high. Consequently, it is important to have the SNR for each model analyte before building the model. This is frequently neglected in many spectroscopic model calibrations, leading to unreliable models. Although these pure compound measurements are required only once, having the pure compound fingerprint from different devices would also allow incorporating differences of various probes and spectrometers.

Second, having the fingerprint of each molecule at various concentrations for a given instrument setup allows in silico spiking of any compound for any process. Although this study was limited to glucose, raffinose, and lactate for one specific CHO-K1 cell culture, we expect this new approach to perform similarly with different cell lines and analytes. The synthetic spiked libraries for specific cell cultures could be easily generated with only a few representative spectra, once a rich library of analytes in water has been generated. Therefore, the experimental burden of this approach is significantly lower, and thus this approach allows reducing time and cost and concomitantly also allows the analytes of interest to be easily extended.

Third, the calibration range can be increased to cover the entire process range or decreased by removing a given known fingerprint concentration of a molecule. As a consequence, the calibration range for data-driven chemometric models can be significantly enlarged. Having the fingerprint of the desired analyte of interest, it is possible to augment existing databases a posteriori. Although the data presented here only covers data from a cell-free supernatant from a perfusion process, the method should be applicable to fed-batch processes with in situ Raman spectroscopy as well. In batch and fed-batch processes, it is often not possible to spike mammalian cell culture processes without altering the process altogether.

Fourth, apart from the standard regression algorithms typically used in chemometric analysis, other new methods (e.g., machine learning methods) may benefit even more from the spectra information enrichment, especially those that require large amounts of training data such as neutral networks. The number of spectra in the SSL grows rapidly with the number of base spectra, concentrations, and analytes measured; hence, large training datasets can be quickly created for specific analytes, cell lines, and media.

Fifth, other approaches, such as just-in-time learning approaches, could also benefit from the SSL approach. As shown in Fig. 7, very few spectra are needed to calibrate robust models within the SSL framework. Thus, if just-in-time learning is combined with the SSL framework, a working model can be created within minutes after the first sampling and updated after each sampling. This methodology is quite attractive as it does not require previous runs to calibrate the models and the models would be process-specific, so a good accuracy/calibration effort ratio would be expected.

Despite the obvious advantages of the novel workflow, high-quality experimental data are of key importance as it still builds the basis of the approach. As the presented work is based on a mammalian perfusion cell culture system, it remains to be shown whether this approach is equally applicable to batch and fed-batch processes, to other bioprocesses using different organisms and other spectroscopic techniques such as infrared spectroscopy. Furthermore, normalizing the process and fingerprint spectra is a crucial step, and we found that dividing by the averaged intensity between 3205 and 3215 cm^−1^, close to the intense symmetric O–H stretching mode of water, proved to be the most effective in our setup. It is worth noting that there are other normalization procedures that may lead to even higher consistencies, particularly under different conditions (e.g., other Raman devices, sensors, or analytes), and this would require testing. Similarly, apart from the standard preprocessing used in this work after generating the SSL (first derivative and second-order polynomial Savitzky-Golay filter with 31 window points), there may be preprocessing methodologies that could improve the accuracy of the models even further. It should also be noted that we currently assume linearity of spectra and analytes, which may not be true in all cases and would require further investigation if this assumption could no longer be justified. Therefore, other machine learning methods should be employed, not only to further improve calibration and robustness but also to identify how many process samples and how many synthetic samples are required to be mixed in order to optimize model building even further. Nevertheless, we strongly believe that the proposed approach is well aligned with regulatory recommendations such as the PAT guidance from the Food and Drug Administration (FDA) or the International Council for Harmonisation of Technical Requirements for Pharmaceuticals for Human Use (ICH) Q2/R1 guidelines, given that our approach enables stable and rigorous mathematical relationships between the spectra and the analytes of interest. Obviously, a proper validation of all steps is deemed necessary in order to apply the proposed approach in a regulatory controlled environment.

## Conclusion

In this manuscript, we have demonstrated the potential of synthetic spectral libraries (SSL) for concentration prediction when combined with standard chemometric approaches. Our findings indicate that the in silico addition of pure compounds provides spectral information comparable to physically spiked measurements. This approach enables the generation of an almost infinite number of information-rich spectra, thereby forming a robust foundation for spectroscopic regression models. It was shown that accurate and robust Raman models can be built within a short timeframe with only a few process samples. This novel method has the potential to enhance the robustness of chemometric models, encompassing various analytes and processes, and might be applicable to other vibrational spectroscopy techniques.

## Supplementary Information

Below is the link to the electronic supplementary material.

Supplementary Material 1 (DOCX 628 KB)

## Data Availability

The data that support the findings of this study are available from the corresponding author upon reasonable request.

## Abbreviations

AOI: Analyte of interest
CHO: Chinese hamster ovary
FDA: Food and Drug Administration
ICH: International Council for Harmonisation of Technical Requirements for Pharmaceuticals for Human Use
IHM: Indirect hard modeling
IR: Infrared
JIT: Just in time
ML: Machine learning
NIR: Near infrared
PLS: Partial least square
PAT: Process analytical technologies
RMSEP: Root mean square error prediction
SNV: Standard normal variate
SSL: Synthetic spectral library
