# Supplementary material for: Synthetic spectral libraries for Raman model calibration
Source: Anal Bioanal Chem. 2025 Jul 8;417(25):5675–89. doi: 10.1007/s00216-025-05985-y (PMC12528253; doi:10.1007/s00216-025-05985-y)
Supplement: Supplementary file 1 — Supplementary Material 1 (DOCX 628 KB) [file 216_2025_5985_MOESM1_ESM.docx]

**Supplementary Information**

**Synthetic Spectral Libraries for Raman Model Calibration**

Louis V. Hellequin^1,2*^, Vicent J. Borràs ^1*^, Patrick Romann^3^, Nandita Vishwanathan^4^, Jonathan Souquet^4^, and Thomas K. Villiger^1^

* These two co-authors contributed equally to this work

^1^ University of Applied Sciences Northwestern Switzerland FHNW, Muttenz, Switzerland

^2^ RWTH Aachen University, Aachen, Germany

^3^ Levitronix, Zürich, Switzerland

^4^ Biotech Process Sciences, Merck KGaA, Corsier-sur-Vevey, Switzerland

Louis V. Hellequin: <https://orcid.org/0009-0004-6712-390X>

Vicent J. Borràs: <https://orcid.org/0000-0002-4728-4042>

Patrick Romann: https://orcid.org/0000-0002-7555-0598

Nandita Vishwanathan: <https://orcid.org/0000-0003-1226-8496>

Thomas K. Villiger: https://orcid.org/0000-0003-0036-2522

Corresponding Author: [thomas.villiger@fhnw.ch](mailto:thomas.villiger@fhnw.ch)

**Table SI 1** DModX values calculated as $\sqrt{\frac{SPE}{K-A}}$, where $SPE$ are the residual sum of squares, $K$ is the number of variables (1250) and $A$ is the number of components (2). The DModX values are obtained using the two PLS models calibrated with the SSL 1 (**Figure 6d**) for glucose and raffinose (**Figure 6e** and **6f**) for the synthetically and physically spiked spectra presented in **Figure 5**. The thresholds are obtained as the 99^th^ percentile of the DModX values obtained for the calibration data.

| **Condition** | **Spiking** | **Glucose model (DModX)** | **Raffinose model (DModX)** |
| --- | --- | --- | --- |
| **+ 8 g/L Gluc.** | Synthetic | 3.7·10^-5^ | 6.4·10^-5^ |
|  | Physical | 3.8·10^-5^ | 8.0·10^-5^ |
| **+ 9 g/L Raf.** | Synthetic | 7.7·10^-5^ | 3.2·10^-5^ |
|  | Physical | 8.4·10^-5^ | 3.2·10^-5^ |
| **+ 4 g/L Gluc.**  **+ 10 g/L Raf.** | Synthetic | 8.8·10^-5^ | 6.2·10^-5^ |
|  | Physical | 8.0·10^-5^ | 5.1·10^-5^ |
| **Threshold**  **(99^th^ percentile)** |  | 9.9·10^-5^ | 1.2·10^-4^ |

**
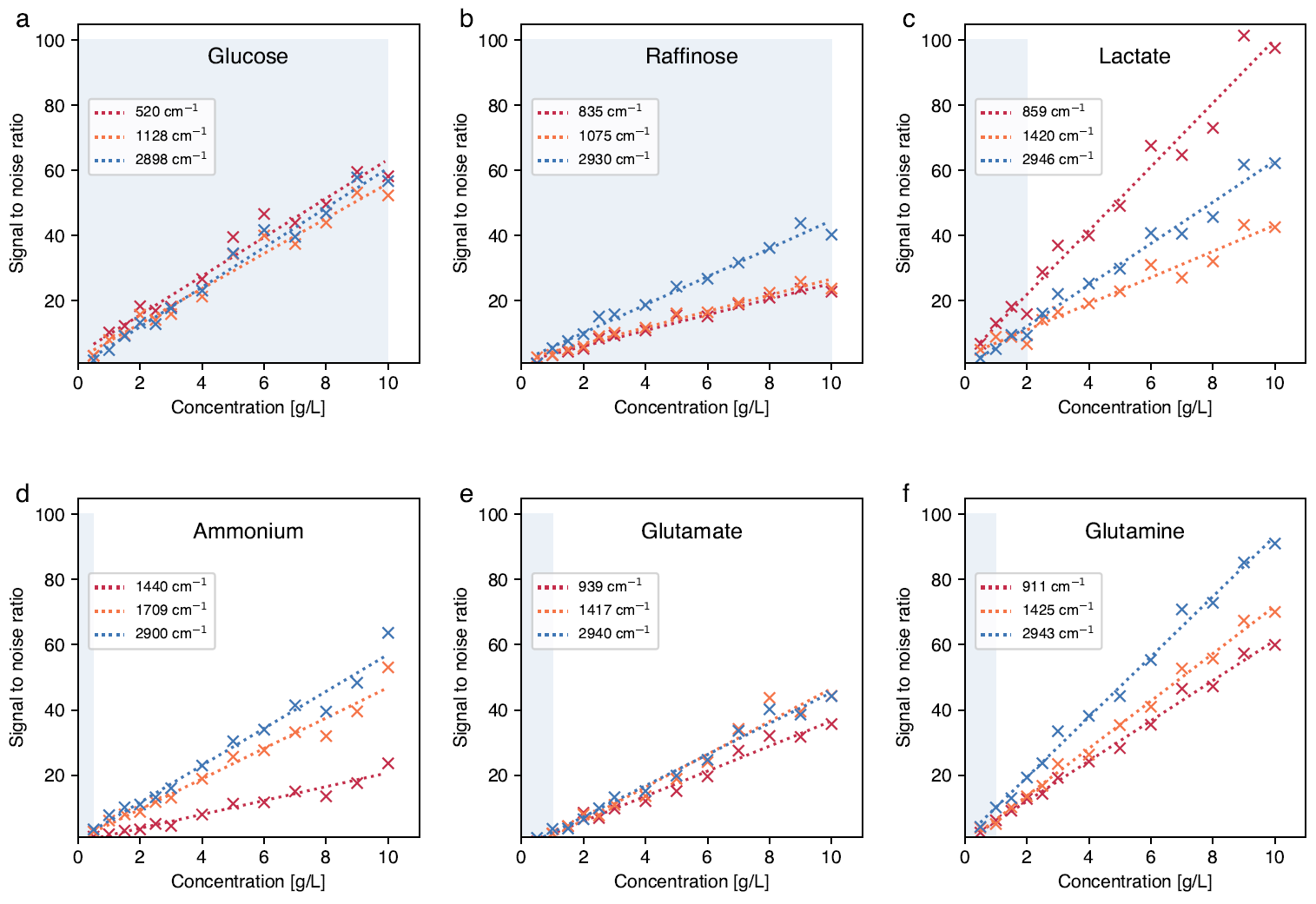
**

**Figure SI 1** Signal-to-noise ratio as a function of the measured analyte concentration


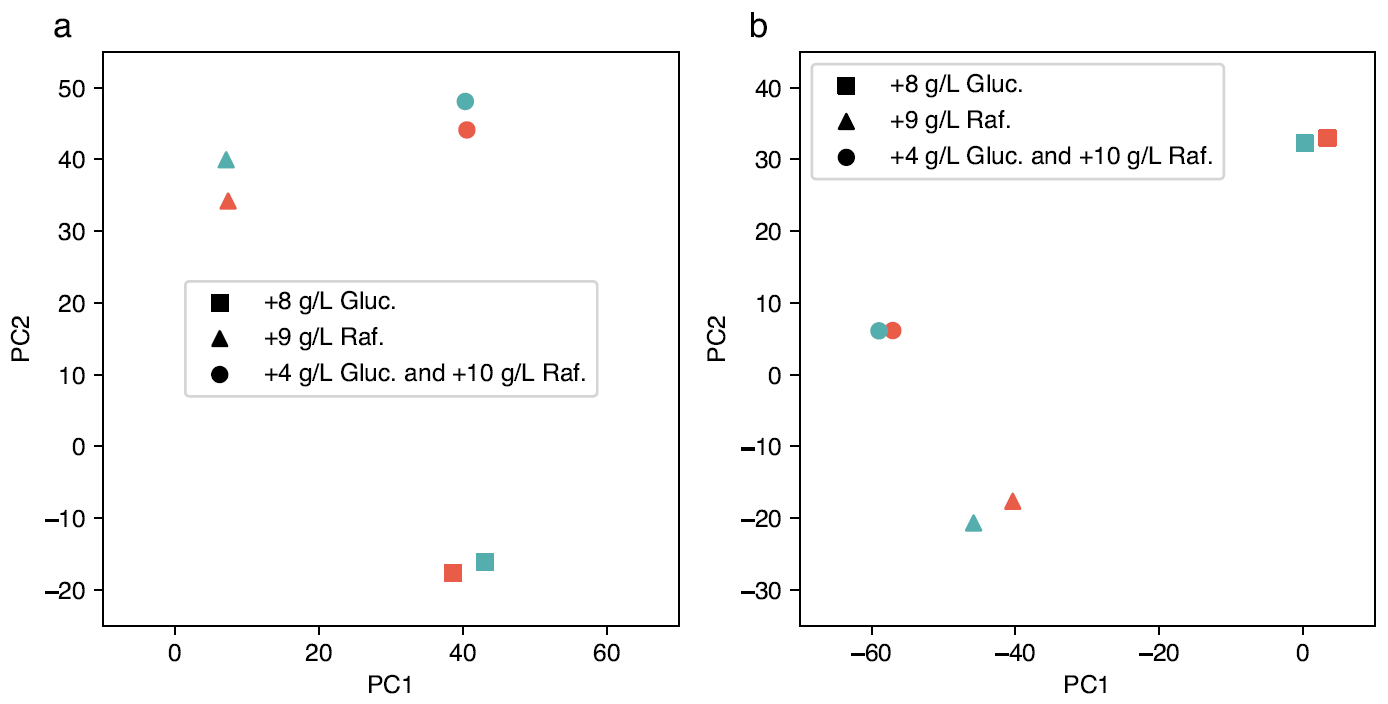


**Figure SI 2 a**) Projection of the synthetically (orange) and physically (dark green) spiked spectra presented in **Figure 5** onto the latent space of the glucose PLS model calibrated with the SSL 1 (**Figure 6**). **b**) Projection of the synthetically (orange) and physically (dark green) spiked spectra presented in **Figure 5** onto the latent space of the raffinose PLS model calibrated with the SSL 1 (**Figure 6**).


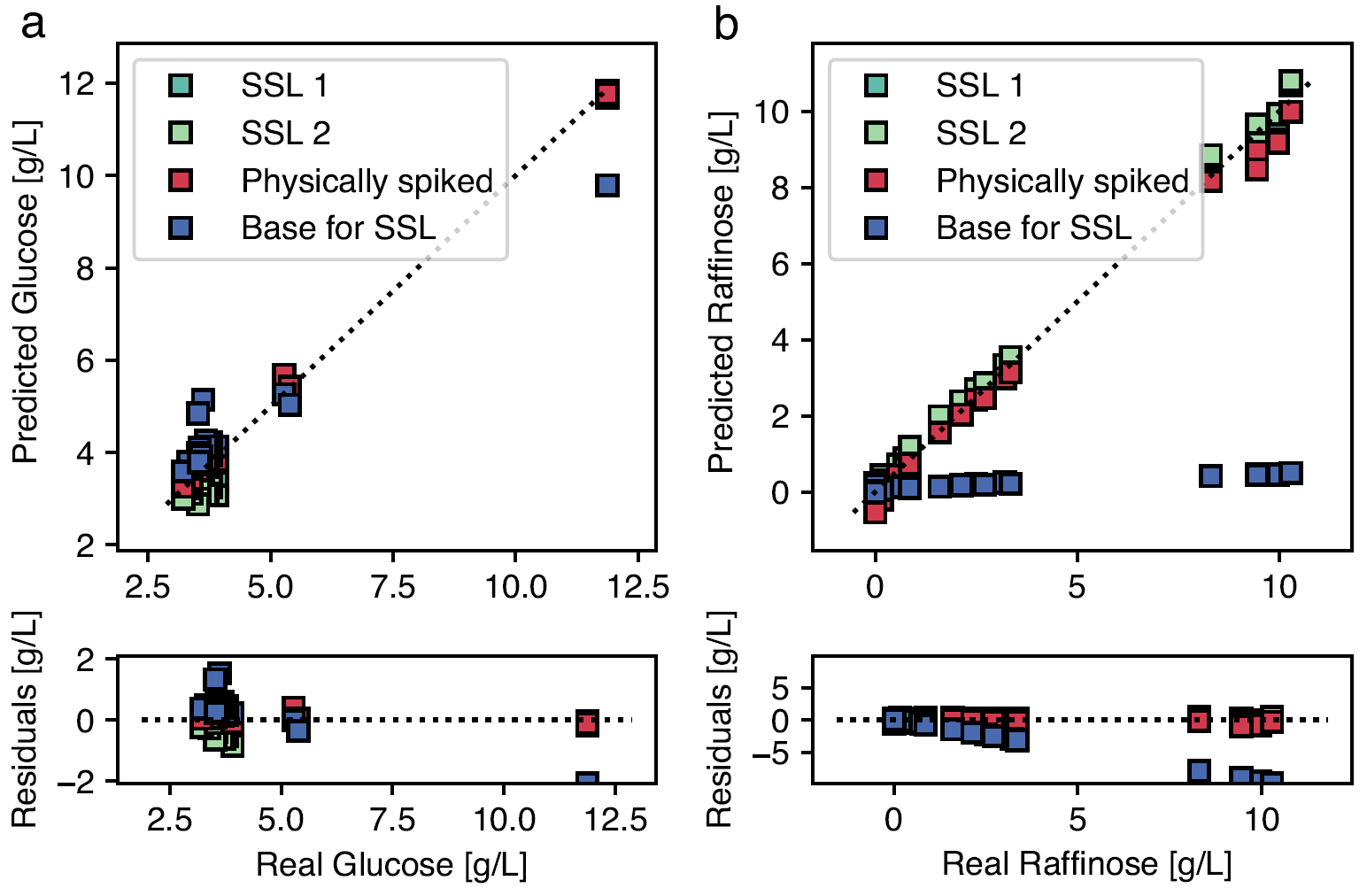


**Figure SI 3 a**) Predicted vs real glucose concentrations for the models calibrated with base (blue), physically (red) and synthetically (light and dark green) spiked spectra. The values correspond to the test run depicted in **Figure 6e**. **b**) Predicted vs real raffinose concentrations for the models calibrated with base (blue), physically (red) and synthetically (light and dark green) spiked spectra. The values correspond to the test run depicted in **Figure 6f**.


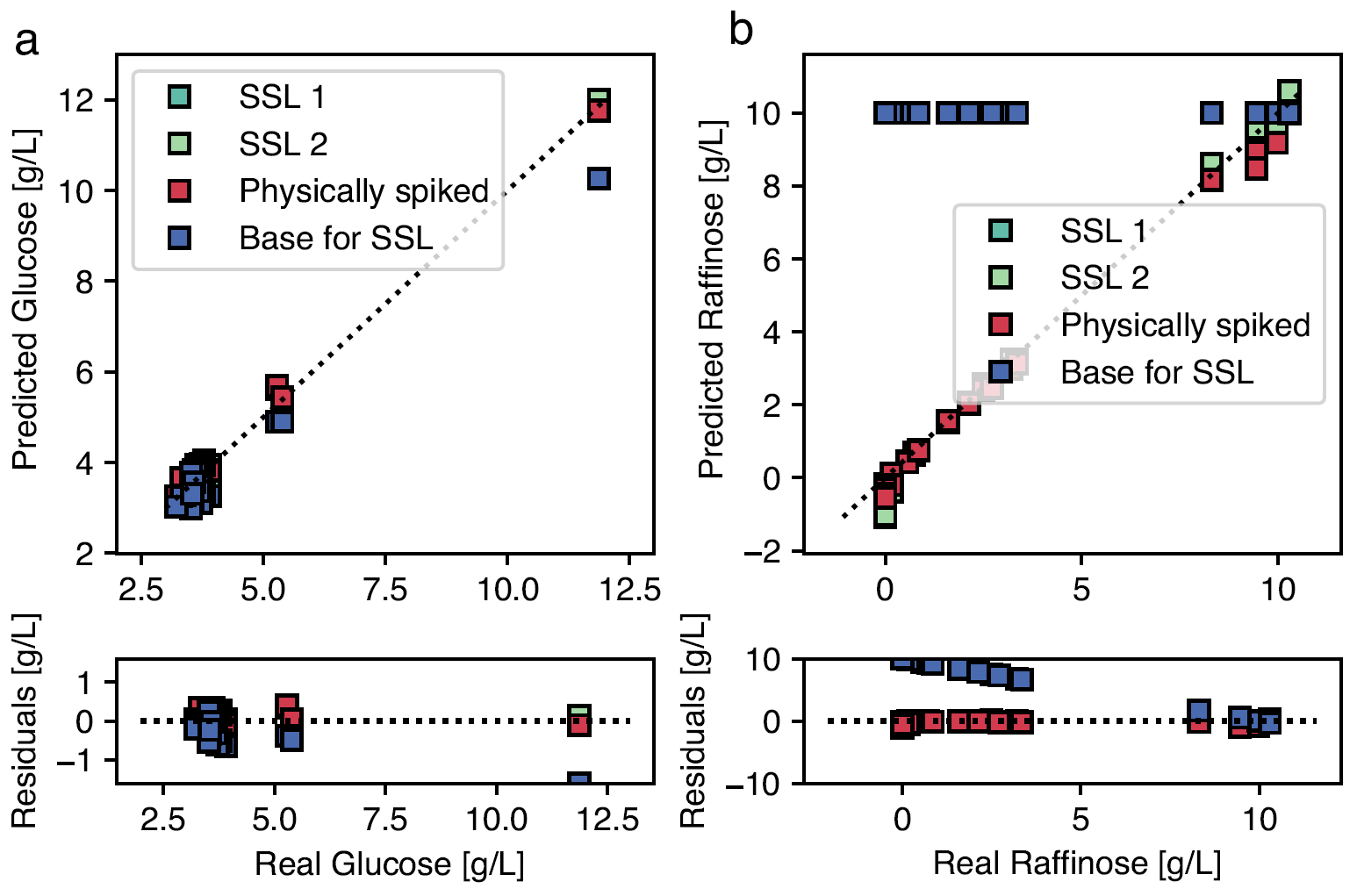


**Figure SI 4 a**) Predicted vs real glucose concentrations for the models calibrated with base (blue), physically (red) and synthetically (light and dark green) spiked spectra. The values correspond to the test run depicted in **Figure 7e**. **b**) Predicted vs real raffinose concentrations for the models calibrated with base (blue), physically (red) and synthetically (light and dark green) spiked spectra. The values correspond to the test run depicted in **Figure 7f**.


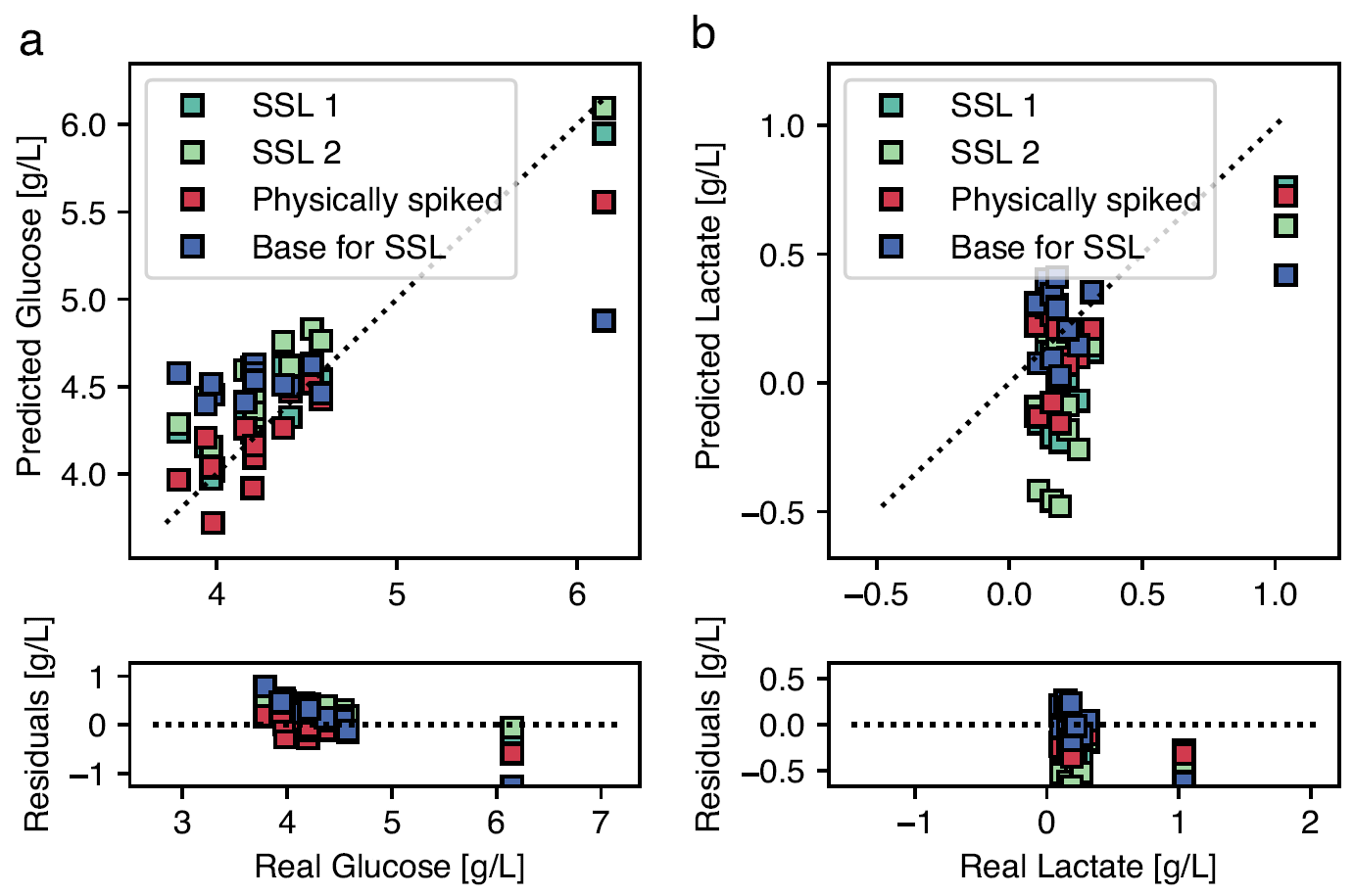


**Figure SI 5 a**) Predicted vs real glucose concentrations for the models calibrated with base (blue), physically (red) and synthetically (light and dark green) spiked spectra. The values correspond to the test run depicted in **Figure 8e**. **b**) Predicted vs real lactate concentrations for the models calibrated with base (blue), physically (red) and synthetically (light and dark green) spiked spectra. The values correspond to the test run depicted in **Figure 8f**.
